# White matter alterations in the internal capsule and psychomotor impairment in melancholic depression

**DOI:** 10.1371/journal.pone.0195672

**Published:** 2018-04-19

**Authors:** Matthew P. Hyett, Alistair Perry, Michael Breakspear, Wei Wen, Gordon B. Parker

**Affiliations:** 1 School of Psychiatry, University of New South Wales, Sydney, New South Wales, Australia; 2 Systems Neuroscience Group, QIMR Berghofer Medical Research Institute, Herston, Queensland, Australia; 3 Centre for Healthy Brain Ageing (CHeBA), School of Psychiatry, University of New South Wales, Sydney, New South Wales, Australia; 4 Metro North Mental Health Service, Royal Brisbane and Women’s Hospital, Brisbane, Queensland, Australia; 5 Black Dog Institute, Prince of Wales Hospital, Randwick, New South Wales, Australia; University of California, San Francisco, UNITED STATES

## Abstract

Emerging evidence suggests that structural brain abnormalities may play a role in the pathophysiology of melancholic depression. We set out to test whether diffusion-derived estimates of white matter structure were disrupted in melancholia in regions underpinning psychomotor function. We hypothesized that those with melancholia (and evidencing impaired psychomotor function) would show disrupted white matter organization in internal capsule subdivisions. Diffusion magnetic resonance imaging (dMRI) data were acquired from 22 melancholic depressed, 23 non-melancholic depressed, and 29 healthy control participants. Voxel-wise fractional anisotropy (FA), radial diffusivity (RD), and axial diffusivity (AD) values were derived for anterior, posterior, and retrolenticular limbs of the internal capsule and compared between groups. Neuropsychological (reaction time) and psychomotor functioning were assessed and correlated against FA. Fractional anisotropy was distinctly increased, whilst RD was decreased, in the right anterior internal capsule in those with melancholia, compared to controls. The right anterior limb of the internal capsule correlated with clinical ratings of psychomotor disturbance, and reduced psychomotor speed was associated with increased FA values in the right retrolenticular limb in those with melancholia. Our findings highlight a distinct disturbance in the local white matter arrangement in specific regions of the internal capsule in melancholia, which in turn is associated with psychomotor dysfunction. This study clarifies the contribution of structural brain integrity to the phenomenology of melancholia, and may assist future efforts seeking to integrate neurobiological markers into depression subtyping.

## Introduction

Melancholic depression has been positioned as the prototypical depressive disorder [1], with a range of proposed neurobiological causes spanning brain structure and function. Clinical studies suggest that melancholia can be subtyped into “structural melancholia”–a late-onset form of the disorder arising from compromised neurovascular functioning—and “functional melancholia”, commencing in adolescence and young adulthood and believed to arise from perturbed brain function. Diffusion magnetic resonance imaging (dMRI) data have been used to highlight white matter abnormalities in both the internal and external capsule in depressed younger and older adults with and without melancholic features [2–5]. All such samples likely include a proportion of ‘true’ melancholic patients, providing support for a functional melancholia class. However, despite increasing evidence for structural brain abnormalities in later-onset melancholia [6] there is a lack of research linking such deficits with behavioral (i.e., neuropsychological, psychomotor) changes in those with functional melancholia.

A convergence of evidence from neuroimaging, genetic and post-mortem studies has highlighted the association between depression and white matter abnormalities [7]. The majority of post-mortem studies—typically of older age individuals—have focused on the prefrontal cortex, with ischemic demyelination [8], and decreases in glial cell size and density [9] observed. The few genetic studies that have been undertaken indicate that genes involved in encoding myelination are decreased in depressed patients [10]. Hickie and colleagues [11] demonstrated that, in older patients with depression, deep white matter lesions predicted regional cerebral blood flow—with the latter being associated with psychomotor slowing as assessed via choice reaction time. Several studies [4,5] have examined white matter integrity in young depressed adults, and identified white matter microstructural changes (reduced fractional anisotropy or FA) of the left anterior limb of the internal capsule. Given the location and associational nature of the internal capsule–where the anterior limb extends from the thalamus, via the basal ganglia, to the frontal cortex—these studies [4,5] provide support for the hypothesis that at least some depressive sub-types are associated with disruptions to the integrity of cortical-subcortical circuitry, and may help explain psychomotor disturbance in those with the melancholic depressive sub-type. For instance, reduced FA values have been reported for internal and external capsule fibers in those with melancholic major depression compared to non-depressed controls [2]. Despite such evidence, previous studies [2–5] have not systematically investigated associations between white matter alterations and functional changes (e.g., neuropsychological function).

Long-range white matter fiber tracts provide a structural backbone for interconnections amongst spatially distributed functional regions of the cortex [12]. The fiber tracts (or ‘bundles’) that form the architecture of these large-scale networks can be probed using dMRI [13]. This imaging approach derives from the restricted diffusion of water in the presence of local white matter bundles, leading to anisotropic diffusion-based signals. White matter structure can hence be assessed *in vivo* in the brain by dMRI sequences that are sensitive to the diffusion of water [14]. Using a simple model of restricted diffusion, namely diffusion tensor imaging (DTI), the integrity of white matter structures can be assessed by the degree of diffusion anisotropy, namely FA. This approach quantifies how variable the diffusion is in different directions, with values ranging from a theoretical low of 0 to a maximum of 1 [15]. Higher values are more likely to be observed in major white matter tracts, whilst values approaching 0 are commonly quantified in cerebrospinal fluid [16]. Other DTI-based quantitative measures include “axial” and “radial” diffusivity (AD and RD). Whilst these latter parameters have been suggested to reflect axonal degeneration (AD) and demyelination (RD) in animal models [17], there may be limitations in inferring underlying tissue structure from tensor values (i.e., AD/RD) in complex pathologies [18]. Despite this caveat, Korgaonkar et al. [2] revealed that melancholic depression was associated with increased RD in the internal capsule. Whether such findings of increased RD reflect demyelination in melancholia is unknown, but at a minimum the inclusion of such parameters, in addition to FA, may assist in clarifying white matter microstructural alterations in the disorder. All parameters (FA, AD, RD) can be computed voxel-wise, allowing for relatively straightforward comparison across subjects. Tract-based spatial statistics (TBSS) is one such method that provides these parameters across spatially distributed white matter pathways [16]. Region of interest (ROI) masks can then be applied to provide region-specific estimates of white matter integrity.

In the present study, we apply TBSS to dMRI data to examine white matter tract organization as reflected by FA, AD and RD across pre-defined ROIs in those with melancholic compared to those with non-melancholic depression and non-depressed healthy controls. Given previous reports highlighting the role of the internal capsule [2–5], we constrain the current analyses to bilateral subdivisions of white matter fibers in this region. Participants also underwent neuropsychological evaluation including assessment of psychomotor function and reaction time. We hypothesized that those with melancholia would show reduced FA in the internal capsule compared to those with non-melancholic depression and healthy control groups. Diffusivity (RD and AD) across sub-regions of the internal capsule was also investigated following previous work highlighting the utility of such parameters in assessing white matter alterations in melancholia [2]. Furthermore, we judged that reduced FA integrity in the internal capsule in those with melancholia would be associated with more impaired clinically rated psychomotor function, and slower reaction times on neuropsychological testing.

## Materials and methods

### Participants

Participants for the current study were drawn from a neuroimaging study of depression undertaken through a specialist depression clinic in Sydney, Australia. Diffusion MRI data were obtained from 22 patients with melancholic depression, 23 patients with a non-melancholic depression and 29 non-depressed control individuals. The age range for inclusion was 18 to 75 years. Depressed participants were deemed eligible if they currently met DSM criteria for a major depressive episode on the Mini-International Neuropsychiatric Interview (MINI [19]). Depressive subtyping was undertaken by clinic psychiatrists according to previously detailed criteria [20]. Features that determined a diagnosis of melancholia included both psychomotor disturbance and distinct anhedonia, and at least five of the following: concentration impairment, mood non-reactivity, anergia, appetite and/or weight loss, early morning wakening, lack of preceding stressors, good previous response to physical (e.g., antidepressant) therapy, and normal personality functioning. Exclusion criteria for all participants included a history of hypomania, mania or psychosis and, for controls, a lifetime history of major depression, as judged by the MINI. Further exclusion criteria included an estimated IQ below 80 on the Wechsler Test of Adult Reading (WTAR [21]), neurological disorder or brain injury, a history of invasive neurosurgery, current and/or past drug or alcohol dependence, and electroconvulsive therapy in the past six months. A formal clinical radiologist report was conducted on structural MRI scans obtained from each participant, and participants were excluded if there was any evidence of cerebrovascular disease, deep white matter hyperintensities or focal lesions. All structural scans were unremarkable and, hence, analyses proceeded on the full sample of 74 participants. An overview of the study was provided to participants prior to obtaining written informed consent. Capacity to consent was determined by assessing clinicians (overseen by senior psychiatrist, GBP) and by the research coordinator (MPH) on the study, in line with the *NHMRC National Statement on Ethical Conduct in Human Research*, *2007* (https://www.nhmrc.gov.au/book/national-statement-ethical-conduct-human-research). No monetary incentive was provided for taking part in the study. The study was approved by the University of New South Wales Human Research Ethics Committee (HREC approval # 08077).

### Clinical and neuropsychological assessment

Depression severity was quantified by the 16-item self-reported Quick Inventory of Depressive Symptomatology (QIDS-SR-16 [22]). The State-Trait Anxiety Inventory (STAI-State and STAI-Trait [23]) was also administered, and overall functioning was assessed using the Global Assessment of Functioning (GAF [24]). The CORE measure [25] was used to assess psychomotor signs, namely non-interactiveness (i.e., cognitive slowing), retardation and agitation, in depressed participants.

Participants completed a computerized neuropsychological battery comprising tests of attention and reaction time (RT), including the Rapid Visual Information Processing (RVP) test, Affective Go/No-Go, and the Intra-Extra Dimensional Set Shift, as taken from the Cambridge Neuropsychological Test Automated Battery (CANTAB [26]). In the current study, we chose to study the white matter correlates of the RVP given it assesses response latency. In the RVP, a white box appears in the center of a computer screen, inside which digits from 2 to 9 appear in a pseudo-random order, at a rate of 100 digits/minute. Participants are asked to detect sequences of digits (2-4-6, 3-5-7, 4-6-8) and, when identified, respond by pressing a button on a response pad as quickly as possible. Averaged RT’s of correct responses to the numerical sequences provide an objective index of psychomotor speed, which, along with CORE ratings of psychomotor impairment was regressed against white matter integrity in different regions of the internal capsule. Neuropsychological data were not available for six (three control, two melancholic, and one non-melancholic) participant(s). Associations between reaction time and FA were analyzed for all other participants.

### Diffusion MRI acquisition

dMRI data were acquired with a 3-T Philips Achieva scanner at Neuroscience Research Australia (NeuRA) in Sydney, Australia, with an 8-channel head coil. One acquisition of 62 directional dMRI data (b = 1000 s/mm^2^ with one non-diffusion-weighted b0 scan) was acquired using a single-shot echo planar imaging (EPI) sequence. The imaging parameters were as follows: TR = 8,897 ms, TE = 68 ms, FOV = 240 × 138 × 240, acquisition matrix size = 96 × 96, flip angle = 90°, 55 slices, slice thickness = 2.5 mm (no gap) yielding 2.5 mm isotropic voxels.

### dMRI preprocessing and tensor calculations

Preprocessing of diffusion images was performed within MRtrix3 (v0.3.12–515; https://github.com/MRtrix3/mrtrix3). Diffusion images from each participant were visualized in FSLView [27] to check for motion artifact, and subjects were removed if any diffusion volumes contained severe geometric distortions or complete slice dropouts [28]. Data from one non-melancholic depressed participant was excluded prior to analysis (leaving 22 in this group). Head motion correction was performed using a custom in-house algorithm [29,30], with rotations of the gradient direction matrix. To decrease spatial intensity inhomogeneities, bias field correction was performed on the b0 image and subsequently applied to all diffusion images [31]. Full description of these steps involved in diffusion preprocessing are described elsewhere [32]. We next applied *dwi2tensor* (MRtrix3) to estimate the tensor coefficients within each brain voxel using a reweighted linear least squares estimator [33]; this was then used to derive maps for FA, and the first (λ1), second (λ2), and third (λ3) eigenvalues.

The TBSS program in FSL [16] was used to transform each subject’s FA image into standard space to calculate tensor values within skeletonized white matter tracts. First, FA maps from each participant were aligned to a 1 × 1 × 1 mm FA target image in MNI standard space. These aligned images were then averaged giving a mean FA image, which was thinned to create a mean tract skeleton. Further thresholding was applied for FA values > 0.2 in order to minimize the partial volume effect and poor registration across participants. Each participant’s FA data were then projected onto the tract skeleton to create a skeletonized FA map. The non-FA images (λ1, λ2, λ3) were projected onto the mean tract skeleton using the same parameters as for the FA images; λ1 values were used to represent ‘axial’ diffusivity, and the average of λ2 and λ3 values represented ‘radial’ diffusivity.

### ROI-based analysis and statistical methods

**Fig 1:**
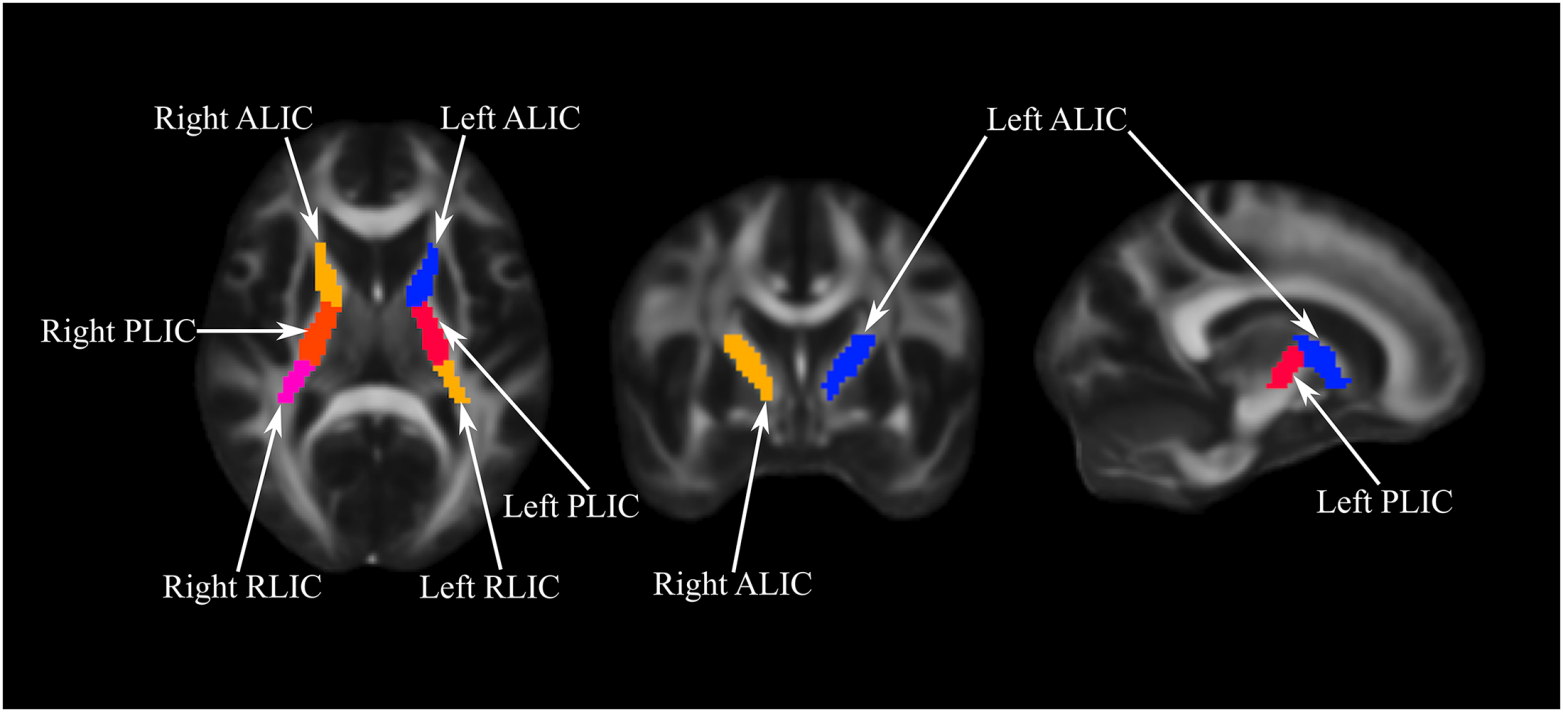
Sub-regions of the internal capsule. ALIC: Anterior Limb Internal Capsule; PLIC: Posterior Limb Internal Capsule; RLIC: Retrolenticular Limb Internal Capsule.

We followed the protocols of the ENIGMA consortium [34] to extract ROI specific information from the skeletonized images. A total of 62 white matter ROI labels were derived from the JHU White Matter Atlas [35]. First, single subject FA, RD and AD values for a given ROI were created using the atlas and skeletonized images. Next, subject-specific data were used to average relevant regions (e.g., left and right internal capsule), generating a mean value weighted by the volumes of those regions. In line with study hypotheses, the current between-group analyses were restricted to six left and right lateralized (non-averaged) ROIs that encompass the internal capsule, namely: the left/right anterior limb, left/right posterior limb, and left/right retrolenticular part of the internal capsule (see Fig 1).

Our analyses principally focus on FA as a measure of local white matter organization in melancholia. Accordingly, group-wise main effects for FA across these regions were analyzed using MANOVA (by fitting a multivariate general linear model, with all regions entered as dependent variables). Following Korgaonkar et al. [2], between group comparisons of RD and AD (using univariate ANOVAs) were only evaluated for regions with significant FA group differences. Significance was set at p ≤ 0.05. We estimated false discovery rate (FDR)-adjusted p-values [36] to control for multiple comparisons. Multiple regression was used to examine the relationship between white matter (FA) integrity and clinical ratings of psychomotor functioning. Partial correlation analysis was employed to analyze associations between FA values in the internal capsule and reaction time performance, with multiple comparisons controlled using FDR correction (significance set at p ≤ 0.05).

## Results

### Demographic and clinical comparisons

The groups did not differ with respect to age, gender or RT on the RVP (Table 1). The age ranges for each group were: melancholic: 22–64 years; non-melancholic: 19–72 years; and control: 20–75 years. The depressed groups did not differ in terms of depression severity as assessed by the QIDS-SR, and anxiety as determined by the STAI-State and STAI-Trait measures. As expected, both depressed groups differed from the healthy control group on both STAI scales, and all groups differed on overall functioning as measured by the GAF scale, with the melancholic group having the lowest scores on this measure. The control group had more years of education and a higher estimated IQ as assessed by the WTAR compared to both depressed groups. Consistent with the primacy of psychomotor disturbances in melancholia, this group had higher total CORE scores, and higher CORE subscale scores, compared to those with non-melancholic depression. Melancholic depressed participants were more likely to be taking medications other than selective serotonin reuptake inhibitors (SSRIs) compared to non-melancholic participants, whilst the latter group was more likely to be taking SSRIs.

**Table 1:** Demographic, clinical and neuropsychological comparisons across melancholic, non-melancholic and control groups.

	Group			Group Comparisons
	Melancholic	Non-Melancholic	Control	
	Mean (SD)	Mean (SD)	Mean (SD)	
**Age**	43.50 (13.17)	39.45 (12.71)	38.34 (13.04)	† t = 1.04, p = 0.31; * t = 1.39, p = 0.17; ‡ t = 0.31, p = 0.76
**CORE—Retardation**	4.86 (3.23)	0.68 (1.78)	NA	† t = 5.32, p < 0.001
**CORE—Non-interactiveness**	3.82 (3.03)	0.45 (1.22)	NA	† t = 4.82, p < 0.001
**CORE—Agitation**	1.09 (1.97)	0.00 (0.00)	NA	† t = 2.59, p < 0.05
**CORE—Total**	9.77 (6.46)	1.14 (2.92)	NA	† t = 5.72, p < 0.001
**Reaction Time—RVP (ms)**	450.40 (119.90)	443.82 (69.75)	426.29 (149.83)	† t = 0.22, p = 0.83; * t = 0.59, p = 0.56; ‡ t = 0.49, p = 0.62
**QIDS-SR**	16.77 (3.41)	14.91 (4.50)	NA	† t = 1.55, p = 0.13
**GAF**	57.27 (8.69)	70.00 (8.02)	94.62 (1.96)	† t = -5.05, p < 0.001; * t = -19.73, p < 0.001; ‡ t = -14.05, p < 0.001
**STAI—State**	50.43 (15.02)	50.36 (12.07)	28.27 (7.44)	† t = 0.02, p = 0.99; * t = 6.18, p < 0.001; ‡ t = 7.47, p < 0.001
**STAI—Trait**	56.81 (12.54)	61.36 (8.96)	32.77 (7.07)	† t = -1.37, p = 0.18; * t = 7.84, p < 0.001; ‡ t = 12.35, p < 0.001
**WTAR**	107.38 (12.33)	107.41 (10.95)	114.92 (11.25)	† t = -0.008, p = 0.99; * t = -2.19, p < 0.05; ‡ t = -2.34, p < 0.05
**Years of education**	14.41 (3.28)	15.15 (3.13)	17.42 (3.58)	† t = -0.75, p = 0.46; * t = -3.02, p < 0.01; ‡ t = -2.25, p < 0.05
**Gender (% female)**	42.9	57.1	57.1	† χ = 1.57, p = 0.21; * χ = 0.00, p = 0.96; ‡ χ = 1.65, p = 0.20
**SSRI (% yes)**	76.9	23.1	NA	† χ = 5.88, p < 0.05
**Drug other than SSRI (% yes)**	75.0	25.0	NA	† χ = 12.57, p < 0.01

^†^melancholic vs. non-melancholic;

* melancholic vs. control;

^‡^ non-melancholic vs. control.

### Internal capsule integrity in melancholia

**Fig 2:**
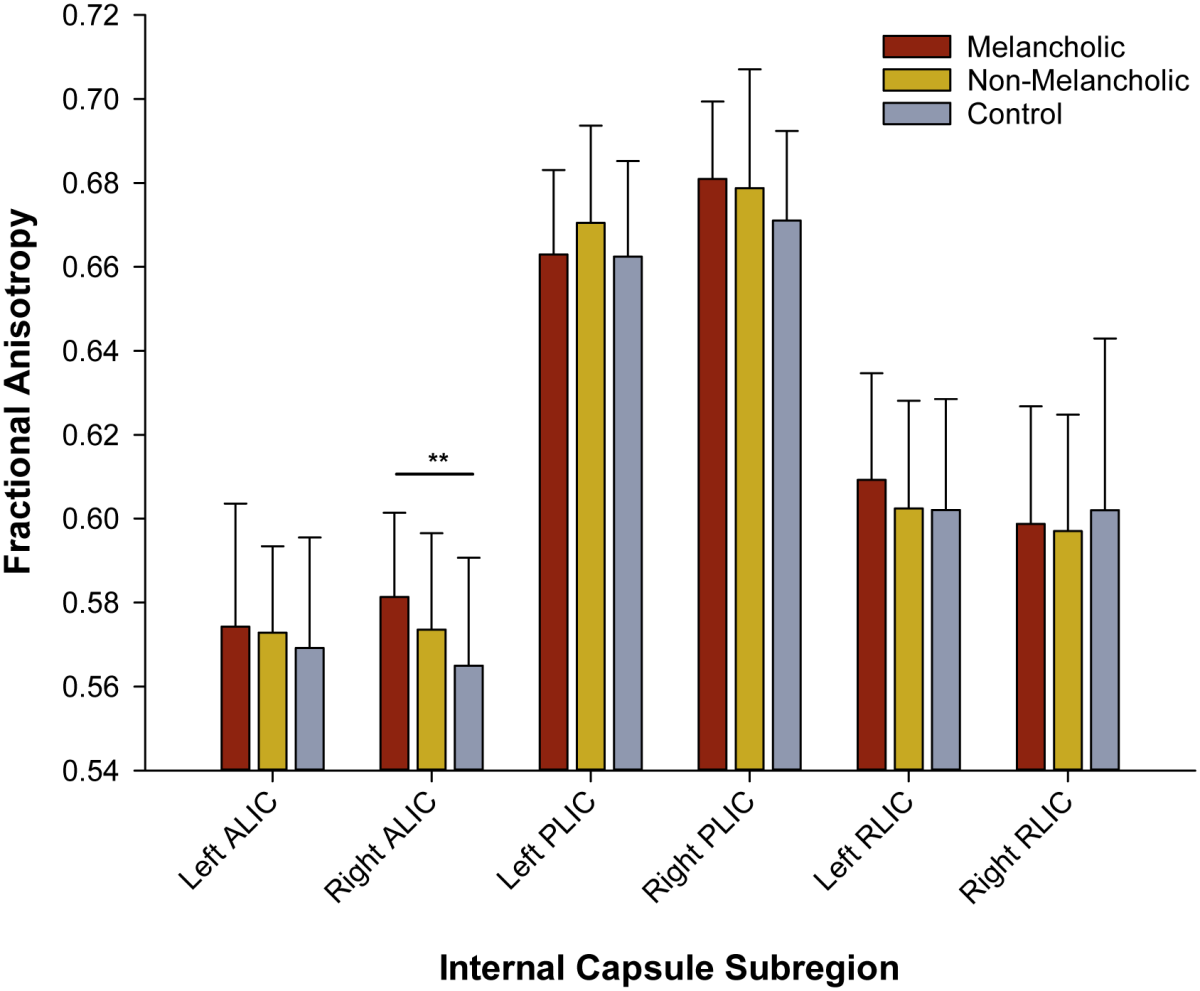
Group differences in fractional anisotropy across internal capsule sub-regions. ** p = 0.024, FDR-corrected.

We applied a multivariate general linear model (MANOVA), controlling for age, to quantify differences in FA between our groups. There was a main effect of group for FA values in the right anterior limb of the internal capsule (F = 4.764, p = 0.012). As illustrated in Fig 2, the melancholic group had higher values in this sub-region (mean = 0.574, SD = 0.029) compared to the control group (mean = 0.569, SD = 0.026) (F = 9.065, uncorrected p = 0.004). This was the only effect to survive FDR correction for multiple comparisons (adjusted p = 0.024). RD values were decreased in those with melancholia (mean = 4.42e-4, SD = 2.03e-5), compared to control participants (mean = 4.58e-4, SD = 2.58e-5) in the right anterior limb of the internal capsule (F = 4.084, p = 0.021). Axial diffusivity did not differ between groups for this region (F = 1.516, p = 0.227). A trend-level effect emerged (before FDR-correction) for FA between the melancholic and control groups for both the right posterior limb (F = 5.37, p = 0.025), with FA increased in this region in those with melancholia. Analyses of diffusivity values for the right posterior limb revealed that the melancholic group had lower RD values (mean = 3.66e-4, SD = 2.19e-5) compared to the control group (mean = 3.82e-4, SD = 2.10e-5) (F = 5.171, p = 0.008). No significant group differences were observed for AD in the right posterior limb of the internal capsule (F = 1.584, p = 0.212). No differences emerged for any region between the non-melancholic and melancholic or control groups.

### Associations between psychomotor change and fractional anisotropy

A multiple linear regression model was fitted to assess whether FA across internal capsule sub-regions, adjusting for depression severity, was predictive of CORE total scores within the melancholic group. The overall model was significant (F = 5.56, p < 0.001). Inspection of individual regression coefficients revealed that, after adjusting for all other sub-regions, FA values in the right anterior limb of the internal capsule were predictive of CORE total scores (t = 2.21, p = 0.030). We next examined the impact of the same predictor variables on CORE subscale scores. The overall model for non-interactiveness was again significant (F = 5.27, p < 0.001), with coefficients for FA values in the right anterior limb, and left and right retrolenticular limbs of the internal capsule reaching significance (all p < 0.05). A similar pattern for the retardation subscale of the CORE was observed (F = 5.24, p < 0.001), with significant regression coefficients observed for the right anterior limb (t = 2.26, p = 0.027). Internal capsule FA, adjusting for depression severity, was not predictive of psychomotor agitation on the CORE scale (F = 1.53, p = 0.172). Further, FA values across internal capsule sub-regions were not predictive of CORE total or subscale scores in the non-melancholic group.

We next analyzed the relationship between psychomotor speed, determined by mean reaction time on the RVP, and internal capsule FA values controlling for depression severity using partial correlations. Within the melancholic group, trend-level effects emerged with associations between FA and RVP mean latency in the right posterior limb (r = 0.439, uncorrected p = 0.060), and the left retrolenticular limb of the internal capsule (r = 0.453, p = 0.052). No associations were observed for the non-melancholic or control groups.

## Discussion

The internal capsule consists of several important white matter fiber bundles of the brain, and is strongly interconnected with a range of cortical and subcortical structures. The anterior limb separates the caudate from the putamen, and relays information from parts of the thalamus to the cingulate and frontal cortices. The posterior limb contains fibers that project sensory information from the thalamus to cortex and, reciprocally, cortico-thalamic connections, and most of the descending fibers to the brain stem and spinal cord, whilst the retrolenticular limb projects from the pulvinar and lateral geniculate nucleus to association and visual cortices [37]. The current study focused on these regions of the internal capsule given their role in motor function—as evidenced in stroke to this region [38]–and the predominance of psychomotor changes in melancholia [1]. We identified *increased* FA, as well as *decreased* RD, in the right anterior portion of the internal capsule. Fractional anisotropy in this region was also correlated with total CORE scores (particularly non-interactiveness). Reduced RD values were also observed in the posterior limb of the internal capsule in melancholia, compared to controls. The subtle increases in FA, and decreased in RD, amongst regions supporting motor function, alongside the observed relationship of increases in FA with increased psychomotor speed, may hence assist in providing insight to the pathophysiology of melancholia.

The specificity of our findings, localized to the right anterior fiber bundle of the internal capsule, may offer a plausible neurobiological explanation for the phenomenology of melancholia. Zou and colleagues [5] reported *decreased* FA in the left anterior limb in patients with major depression, whilst Korgaonkar and colleagues [2] also identified decreased FA across a constellation of regions including the retrolenticular limb of the internal capsule in those with melancholia. This is consistent with several other previous studies reporting decreases in FA across a range of brain regions in major depression [4,7]. Previous work has also identified *increased* RD in subregions of the internal capsule in those with major depression with [3] and without [2] melancholic features. The current analyses demonstrate the reverse pattern—instead of decreased FA and increased RD we here provide evidence that melancholia is associated with *increased FA* and *decreased RD* across specific internal capsule regions. Several potential explanations for these contrasting findings can be highlighted. Patients in the previous studies were diagnosed with major depressive disorder (with and without melancholic features), which may have contributed to the differential findings. Our patient groups were initially diagnosed with major depressive disorder using DSM criteria, and then clinically categorized as either melancholic or non-melancholic by psychiatrists using previously detailed criteria [20,39]. These clinical decision rules included judging the severity and persistence of psychomotor impairment—amongst other features—with its presence, along with depressed mood and affect, indicative of a melancholic diagnosis. Hence, we judge that those included with ‘melancholia’ in previous studies may have included many with a true non-melancholic depression so influencing results. The specificity of the current findings, in more refined groups, offers some clarification of white matter integrity of the internal capsule in melancholic depression. This significant increase in FA in the right anterior limb aligns with studies of those with schizophrenia, which is characterized by a global decrease in FA, but with focal increases in specific regions suggested to underpin symptom generation [40]. Increased FA in bilateral regions of the anterior limb of the internal capsule has also been reported in obsessive-compulsive disorder [41]. It has also been suggested that FA is generally negatively correlated with RD [42]. The current findings align with this rule, as well as previous work demonstrating increased FA, and decreased RD, in the left superior longitudinal fasciculus (inferior temporal cortex) in bipolar depression [43], which is typically melancholic in nature [44]. These lines of evidence indicate that there is no simple benchmark for interpreting FA (or RD/AD) values across psychiatric diagnostic sub-groups. That said, however, and by exercising a degree of caution, inferences can be made as to the role of white matter integrity in melancholia and its phenomenology.

It has been noted that it is difficult to determine what FA and diffusion parameters measure with regards to white matter microstructural “integrity” [45]. Changes in FA are widely interpreted as speaking to white matter “integrity” although, in the absence of a specific white matter disorder, caution has been raised in these regards [46]. Furthermore, one cannot say whether group differences in diffusion are a result of disrupted axonal processes or fluctuations in myelination [18]. For example, FA can be observed in myelin deficient model systems (e.g., rat brain) [47]. Despite this, in interpreting diffusion processes, myelin likely plays a supporting role to axonal membrane integrity [45]. The increased FA values in the anterior limb in melancholia compared to controls may reflect activity-dependent strengthening of axonal membrane fibers. Whether this is an etiological ‘mechanistic’ process contributing to key features of melancholia is not known, but, if at all causal, may be related to psychomotor disturbance in melancholia given the observed relationship with increased reaction times. The current results also extend upon previous findings that identified dysconnectivity between insula- and attentional control-based neuronal systems in melancholia compared to both healthy control participants and those with non-melancholic depression [39]. A previous post-mortem analysis of insula anatomy has found that bilateral internal capsule fibers provide a landmark for the anterior border of bilateral insula cortices [48]. It may be that the reported dysconnectivity between insula and frontoparietal (attentional) systems in melancholia is at least partially driven by disruptions to ascending white matter pathways linking subcortical and cortical brain structures. That said, a number of white matter pathways pass through the insula, including the superior longitudinal fasciculus [49]. Consideration of the distributed architecture of such fibers will hence be important when attempting to clarify relationships between white matter pathway integrity and neuronal interactions across brain regions.

Several study limitations can be noted. Whilst relationships were observed between psychomotor function and internal capsule integrity in the melancholic group, it is not possible to determine whether such findings are indicative of the cause of melancholia and its symptoms. Studying a larger cohort in a longitudinal setting would help clarify whether white matter disruptions are indeed implicated in the etiology of melancholia. A larger cohort would also allow testing as to whether other disorder-specific features, such as abulia and anhedonia, are related to white matter microstructure deficits. Our study groups also differed in their medication regimes, which may have influenced the results. It is unlikely that recruitment of an unmedicated sample of melancholic depressed patients could be achieved—given the inherent severity of the condition—but specificity would be improved if groups were matched for medication class. It would also be useful to examine the impact of a broad spectrum of medications and other treatment modalities (e.g., direct current stimulation and electroconvulsive therapy) on change in diffusion anisotropy in subtypes of depression, as recently explored in a treatment prediction study in major depression [50].

## Conclusions

We demonstrate that white matter alterations observed in melancholia are significantly associated with a key feature of the disorder, namely psychomotor slowing. It is suggested that the observed increases, rather than previously reported decreases, in FA (as well as attendant decreases in RD) in the internal capsule are specific to melancholia and may reflect disruptions of willed action. The current findings hence assist in clarifying the neurobiology of this depressive sub-type, in positioning melancholia as a distinct categorical entity, and in refining biomarkers of specific depressive phenotypes as psychiatry seeks to reshape classificatory frameworks based on shared and unique neurobiological mechanisms amongst its disorders [51].
